# Identifying Emergency Department Symptom-Based Diagnoses with the Unified Medical Language System

**DOI:** 10.5811/westjem.2019.8.44230

**Published:** 2019-10-24

**Authors:** Benjamin H. Slovis, Danielle M. McCarthy, Garrison Nord, Amanda MB Doty, Katherine Piserchia, Kristin L. Rising

**Affiliations:** * Sidney Kimmel Medical College at Thomas Jefferson University, Department of Emergency Medicine, Philadelphia, Pennsylvania; † Northwestern University Feinberg School of Medicine, Department of Emergency Medicine, Chicago, Illinois; ‡ Sidney Kimmel Medical College, Thomas Jefferson University, Philadelphia, Pennsylvania

## Abstract

**Introduction:**

Many patients who are discharged from the emergency department (ED) with a symptom-based discharge diagnosis (SBD) have post-discharge challenges related to lack of a definitive discharge diagnosis and follow-up plan. There is no well-defined method for identifying patients with a SBD without individual chart review. We describe a method for automated identification of SBDs from ICD-10 codes using the Unified Medical Language System (UMLS) Metathesaurus.

**Methods:**

We mapped discharge diagnosis, with use of ICD-10 codes from a one-month period of ED discharges at an urban, academic ED to UMLS concepts and semantic types. Two physician reviewers independently manually identified all discharge diagnoses consistent with SBDs. We calculated inter-rater reliability for manual review and the sensitivity and specificity for our automated process for identifying SBDs against this “gold standard.”

**Results:**

We identified 3642 ED discharges with 1382 unique discharge diagnoses that corresponded to 875 unique ICD-10 codes and 10 UMLS semantic types. Over one third (37.5%, n = 1367) of ED discharges were assigned codes that mapped to the “Sign or Symptom” semantic type. Inter-rater reliability for manual review of SBDs was very good (0.87). Sensitivity and specificity of our automated process for identifying encounters with SBDs were 84.7% and 96.3%, respectively.

**Conclusion:**

Use of our automated process to identify ICD-10 codes that classify into the UMLS “Sign or Symptom” semantic type identified the majority of patients with a SBD. While this method needs refinement to increase sensitivity of capture, it has potential to automate an otherwise highly time-consuming process. This novel use of informatics methods can facilitate future research specific to patients with SBDs.

## INTRODUCTION

Patients are commonly discharged from the emergency department (ED) without a pathological diagnosis to explain their symptoms, with one study finding that over one third of patients leave the ED with a symptom-based diagnosis (SBD).^1^ Studies exploring reasons for return ED visits have identified high levels of patient uncertainty related to lack of a definitive diagnosis as one cause for return.^2^–^4^ These findings suggest the need for further research regarding the impact of and needs associated with receiving a SBD at the time of ED discharge and on patient transitions home from the ED. Research on this topic is challenging, however, because electronic health records (EHR) do not have a unique identifier for SBDs, and there is no agreed upon classification system for these conditions. This leaves manual chart review as the primary option for identifying these patients,^5^ which is a highly subjective and time-consuming process.

The Unified Medical Language System (UMLS) is a compilation of multiple biomedical vocabularies that facilitates interoperability between information systems.^6^ The UMLS consists of three main components: the Metathesaurus; the Semantic Network; and the SPECIALIST Lexicon.^7^ The UMLS Metathesaurus is a biomedical thesaurus that connects and organizes over 200 vocabularies into unique concepts, allowing varying terms for the same concept to be linked together so that relationships can be established between different concepts. For instance, the *International Statistical Classification of Diseases and Health Related Problems*, 10^th^ edition (ICD-10)^8^ code “R07.4 – Chest Pain” and Systematized Nomenclature of Medicine – Clinical Terms (SNOMED-CT)^9^ code “29857009 – Chest Pain (finding)” both map to the UMLS concept unique identifier (CUI) “C0008031 – Chest Pain.”

The Semantic Network is a series of semantic types that more broadly categorize concepts in the Metathesaurus and allow for relationships between these concepts.^7^ For example, the UMLS concept for chest pain and headache (C0018681) both map to the semantic network identifier “T184 – Sign or Symptom.”

Finally, the SPECIALIST Lexicon is a biomedical dictionary of English terms used for natural language processing (NLP). Each entry contains syntactic, morphological, and orthographic information for a term, as well as acronyms and abbreviations. This allows unification of different variations of the same term that would usually be documented in text in multiple forms (eg, “testing,” “tested” and “test” are all treated as the same verb “test”). For instance, a term search for “chest pain” returns its base term, spelling variant (chest-pain), identification number, syntactic category (noun), and variants describing it as both a countable noun (“I’m having chest pains”) and uncountable noun (“the most common complaint was chest pain”). A search for “CP” (a common acronym for “chest pain”) returns multiple entries including the noun entry for “chest pain.”

The UMLS has previously been used to facilitate ED-based research. Metzger et al. used it to develop an automated process to identify suicide attempts in the ED. For this process, they used NLP to assign codes from five different terminologies to medical terms written in natural language, and then used the Metathesaurus to identify similar concepts between the different terminologies.^10^ Travers et al. evaluated the UMLS as a foundation for the generation of an ED chief complaint (CC) vocabulary.^11^ Lu et al. used the UMLS to map ED CCs to UMLS concepts for the purpose of grouping CCs into syndromic categories to allow for automated monitoring of disease outbreaks.^12^ Finally, Doan et al. used the UMLS to construct a lexicon of terms from ED documentation that identifies patients who should be considered for a diagnosis of Kawasaki disease.^13^ To our knowledge, the UMLS has not yet been used to identify cohorts of patients based on categories of ED discharge diagnoses for use in research.

**Population Health Research Capsule**

What do we already know about this issue?

*Patients discharged from the emergency department with a symptom-based diagnosis (SBD) commonly experience post-discharge challenges. There is no automated process to identify SBDs*.

What was the research question?

Can an automated and accurate process to identify SBDs be developed?

What was the major finding of the study?

*Our automated process to identify SBDs had high sensitivity and specificity compared to the gold standard of manual review*.

How does this improve population health?

*Development of an automated, accurate process to identify SBDs would facilitate how we understand the primary needs and barriers of patients discharged with an SBD*.

In our current research, we sought to engage patients who had recently been discharged from the ED with a SBD via follow-up interviews. In previous work, these patients were identified manually. Here we describe the process by which we mapped patients’ ED diagnoses to UMLS concepts to extract the semantic type for each diagnosis, thus generating a list of patients recently discharged from the ED with a likely SBD. The primary goal of this study was to compare this automated process of identifying SBDs to the “gold standard” of manual review.

## METHODS

### Study Design, Setting and Population

We performed a retrospective data analysis on data from the EHR at a single, urban, academic hospital. These methods were approved by the hospital institutional review board. The hospital had over 68,400 ED visits the year prior to this study with approximately 64% of patients being discharged from the ED. The process we designed was to identify all adult patients (18 years and older, non-pregnant) who were discharged from our ED with a SBD within a 30-day period. Exclusion criteria included any patient who did not receive an ED disposition of discharge (ie, left against medical advice, transfer, admission to inpatient or observation status), and any patient who did not have a discharge diagnosis assigned.

### Data Collection and Processing

We first queried documentation from the hospital’s EHR system Epic (Epic Systems Corporation, Verona, WI) via a third-party analytics software Qlik Sense (Qlik, Radnor, PA) to develop a list of all potentially eligible patients from May 2018. At the time of discharge, physicians enter a “clinical impression,” which is derived from a local vocabulary linked with an ICD-10 based diagnosis code in the ED. We extracted the primary ICD-10 code and the associated “primary clinical impression” of the discharge diagnosis for each encounter to generate a list of potentially eligible patients. In cases for which there were multiple codes assigned, we used the first diagnosis code.

We downloaded the full release of the 2018AA UMLS^14^ and created a custom subset of ICD-10 Clinical Modification via Metamorphosys,^7^ the UMLS installation and customization program. Complete instructions on the installation of Metamorphosys are described by the U.S. National Library of Medicine.^15^ We read the UMLS Rich Release Format (RRF) files for codes (MRCONSO.RRF) and the semantic types (MRSTY.RRF) into R statistical software v 3.3.2 (R Core Team, Vienna, Austria).

We then read into R the list of ICD-10 diagnosis codes and the associated discharge diagnosis associated with our study population. We used the package “data.table” v 1.11.4 (Matt Dowle and Arun Srinivasan) to map ICD-10 codes to their respective UMLS CUIs from MRCONSO (excluding term types deemed suppressible) and mapped the resulting CUIs to their appropriate semantic type from MRSTY. We isolated the unique relationships between ICD-10s, CUIs and semantic types, and linked these to each ICD-10 included in our study population.

This resulted in a table consisting of ICD-10 codes, associated discharge diagnoses, CUIs, and associated semantic types. For example, the ICD-10 “R68.2” is associated with the diagnosis of “Dry mouth” which mapped to the CUI: “C0478155 – Dry mouth, unspecified” which holds the semantic type “T184 – Sign or Symptom.”

### Data Analysis

For comparison, two authors (KLR and DMM) independently reviewed each discharge diagnosis and their respective ICD-10 code while blinded to the mapped semantic type, and categorized each diagnosis as either a SBD or non-SBD electronically in a spreadsheet. We calculated Cohen’s kappa for inter-rater reliability. In the event of a disagreement, a third author (BHS) performed review to resolve the discrepancy.

The results of the manual categorization were linked to the output of the UMLS mapping. We calculated frequencies for each combination of ICD-10 code, discharge diagnosis, CUI, semantic type, and SBD category. Using the manual categorization as the “gold standard,” we also calculated sensitivity and specificity of the UMLS mapping to the “Sign or Symptom” semantic type. We focused specifically on mapping to the semantic type “Sign or Symptom,” as this was determined by the team to be the semantic type that should logically contain SBDs.

We calculated the statistical outcomes twice. The first analysis was conducted at the level of the patient encounter, which applies clinically to the question of whether each patient was discharged with a SBD. The second analysis was conducted at the level of the discharge diagnosis, thus assessing whether each unique diagnosis that was provided across one or more encounters was a SBD. We mapped all primary discharge diagnosis codes to CUIs in the Metathesaurus and their associated semantic types from the Semantic Network for each CUI. Our EHR uses a proprietary discharge diagnosis dictionary where multiple discharge diagnoses can be assigned the same ICD-10 code. Therefore, there are multiple synonyms within our discharge dictionary, and a high number of diagnoses could map to a small number of ICD-10 codes. For instance, “Seizure (CMS/HCC [Centers for Medicare and Medicaid Services/hierarchical condition category])” and “Seizures (CMS/HCC)” are separate diagnoses in our dictionary that only differ in plurality, but are both associated with the same ICD-10 code “R56.9.”

## RESULTS

A total of 5705 patients visits occurred in our ED during the study period, out of which we identified 3879 (67.9%) that received an ED disposition of discharge. Of these, 237 (6.1% of discharges) met exclusion criteria resulting in 3642 (63.8 % of all visits) eligible ED discharge visits that were included in our patient encounter level analysis. Of these, 53.1% were for female patients with a median age of 41 years (interquartile range [IQR] 28–57 years) and 46.9% were for male patients with a median age of 43 year (IQR 31–56 years). These 3642 patient encounters received 1382 unique discharge diagnoses that we included in our discharge diagnosis-level analysis. These discharge diagnoses corresponded to 875 unique ICD-10 codes that mapped to 873 unique CUIs associated with 10 unique semantic types. Inter-rater reliability for the manual categorization of discharge diagnoses as SBD or non-SBD was very good at 0.87, with discrepancy in 73 (5.3%) diagnoses.

### Patient Encounter Level Results

Of the 3642 patient encounters that resulted in discharges, there were 1367 encounters (37.5% of ED discharges) assigned a “Sign or Symptom” semantic type by our software (Table 1).

When applying the results of our manual review to the full dataset of discharge encounters, we identified 1288 patient encounters with a discharge diagnosis categorized as a SBD by manual review and assigned a semantic type of “Sign or Symptom.” There were 79 encounters with discharge diagnoses not categorized as SBDs but assigned the semantic type of “Sign or Symptom.” There were 2042 encounters with a discharge diagnosis code not assigned the semantic type of “Sign or Symptom” and also not categorized as SBDs. There were 233 encounters that were not assigned the semantic type “Sign or Symptom” but categorized as SBDs in our manual review. Therefore, when examining all discharge encounters in our dataset (ie, examining the accuracy of our software for identifying SBDs on the level of the patient), our methods resulted in a sensitivity of 84.7% (95% confidence interval [CI], 82.8 – 86.5) and a specificity of 96.3% (95% CI, 95.4 – 97.0). Positive predictive value was 94.2% (95% CI, 92.9 – 95.3) and negative predictive value was 89.8% (95% CI, 88.6–90.8). These results are presented in Table 2. The top 10 diagnoses, ICD-10 codes, and frequencies for each grouping of semantic type assignment and SBD category at the encounter level are displayed in Tables 3–6.

### Discharge Diagnosis Level Results

A total of 1382 unique discharge diagnoses were associated with the 3642 ED discharge encounters. Of these diagnoses, 314 (22.7%) were assigned the semantic type of “Sign or Symptom” by our software. With manual review, we identified 369 (26.7%) diagnoses as a SBD. When comparing the semantic types assigned by the software to those categorized as a SBD by manual review, 277 of the unique discharge diagnoses assigned “Sign or Symptom” were categorized as a SBD, while the other 37 assigned “Sign or Symptom” were not categorized as a SBD.

There were 976 unique discharge diagnosis codes not assigned the semantic type “Sign or Symptom” that were also not categorized as SBDs, and 92 diagnosis codes not assigned the semantic type “Sign or Symptom,” but categorized as SBDs in our manual review. Therefore, when examining the accuracy of the software for identifying SBDs by classifying diagnoses to the semantic type of “Sign or Symptom,” our methods resulted in sensitivity of 75.1% (95% CI, 70.3–79.4) and a specificity of 96.4% (95% CI, 95–97.4) with a positive predictive value of 88.2% (95% CI, 84.4 – 91.2) and a negative predictive value of 91.4% (95% CI 89.9 – 92.7). A 2 × 2 table of these results is presented in Table 7.

## DISCUSSION

We describe a novel automated electronic approach using the UMLS to identify groups of patients who have been discharged from the ED with a SBD (ie, “shortness of breath”) instead of a disease-specific diagnosis (ie, asthma exacerbation). Using manual physician review as the “gold standard,” we demonstrated a high sensitivity and specificity for the identification of SBDs using the UMLS semantic type of “Sign or Symptom.”

The UMLS has been used in prior studies on ED EHR data for purposes including epidemiologic surveillance, constructing chief complaint dictionaries, and automated screening of rare conditions.^10^–^13^ These applications typically use UMLS with NLP, where free text is analyzed (eg, provider notes) for concepts that were not otherwise captured in the EHR. Our work is different in that it was not intended for use with NLP or decision support, but rather was focused on automating the categorization of data fields that are not disease-specific for the purpose of identifying patients for research.

Our recent work suggests that many patients discharged from the ED with a SBD have struggles related to their lack of a definitive diagnosis, with further work needed to explore the challenges unique to this patient population.^3^,^4^,^16^–^18^ Until now, there has not been a well-defined automated process for identifying these patients based upon their category of diagnosis (ie, “symptom-based”) instead of a specific diagnosis name (eg, “myocardial infarction”). Our software was able to identify SBDs with a high sensitivity and specificity on the encounter level. False positives (assigned “Sign or Symptom” but not categorized as SBD) generally appeared to be pain or neurologic syndromes such as “seizure” and “musculoskeletal pain.” Some of these diagnoses are inherently ambiguous, as there are both primary conditions and secondary causes for many of these diagnoses.

False negatives (not assigned “Sign or Symptom” but categorized as a SBD) appear from predominantly three semantic types: “Finding,” “Disease or Syndrome” and “Pathologic Function.” Further refinement of our software may reduce the frequency of false negatives as we believe many of these diagnoses, such as “acute left ankle pain” or “vaginal discharge,” could also be described as a “Sign or Symptom.” However, it is important to note that the sensitivity of our analysis significantly improved (84.7% vs 75.1%) when examining our results on the more clinically-relevant patient encounter level, as opposed to the diagnosis level.

This work informs both future retrospective research that requires identification of this patient population, as well as potential future prospective work to identify and intervene on these patients in real time. Future integration of semantic types with ED discharge diagnoses could allow for automation of this process in real time, building the foundation for decision-support systems that guide providers to avoid SBDs or to provide additional assistance to patients discharged with a SBD.

## LIMITATIONS

Our analysis was limited to a single academic institution that uses a single EHR. Our implementation design includes ICD-10 codes associated with clinical diagnoses made in the ED; however, other hospital systems may use other medical terminologies or proprietary diagnosis dictionaries. The UMLS allows for various search modes, including various terminologies, ontologies and search terms; however, a comparison of these methods is needed to ensure reliable results.

In addition, even among institutions using similar EHRs and impressions mapped to ICD-10, there are likely to be health system and regional variation in practice patterns for the level of detail provided at the time of discharge (eg, gastroenteritis vs vomiting and dehydration), which may make these methods less reliable. For the purpose of this analysis we used the first diagnosis and associated ICD-10 code assigned to each patient encounter, which is defined as the “primary clinical impression” in our EHR. We presume that the “primary clinical impression” is the diagnosis made by the treating provider most closely associated with the patient’s encounter.

The analysis of additional diagnoses assigned at the time of treatment and the development of a process to weigh the value of combinations of SBDs and non-SBDs were outside the scope of this research. It is possible that if a patient was assigned additional diagnoses that were not SBDs, their overall level of uncertainty could be lower or vice-versa. Further analysis will have to be performed to include additional diagnosis codes and develop a process to determine the level of uncertainty associated with combinations of SBDs and non-SBDs. Also, we mapped ICD-10 codes to the first CUI returned by the UMLS. It is possible that additional CUIs could be more appropriate in certain cases, although an analysis to compare various CUIs would deviate significantly from the simple methods described in this manuscript.

We used manual review and categorization of discharge diagnoses by two emergency physicians (with a third as an arbitrator) as the gold standard for SBDs. While our reviewers had high inter-rater reliability (0.87), they were not blinded to the goals of the study, and may have been biased in their categorization of SBDs. Additionally, as noted above, some of these discharge diagnoses are inherently ambiguous. Our team of raters established the list of SBDs via consensus and in these ambiguous cases attempted to consider the case from the viewpoint of the patient. For example, if a patient presents with pain in a limb, they are often concerned about a fracture or sprain; in this case, receiving a diagnosis of musculoskeletal pain (while still ambiguous and less specific than “sprain” or “contusion”) has more specificity than the presenting complaint of “leg pain.” In contrast, when a patient presents unable to urinate and is discharged with a diagnosis of “urinary retention,” they have gained no specificity beyond that with which they presented. It was this sort of rationale that informed our decision-making and why “musculoskeletal pain” is not considered a SBD, but “urinary retention” is.

However, despite our high inter-rater agreement, we acknowledge that others, including both patients and medical professionals, may disagree with our determination of SBD classification. Future work is needed to refine this method before routine use to identify complete cohorts of patients or to assess frequencies of occurrence. Further, by categorizing SBDs, we are not attempting to assign value to the SBD or encouraging emergency physicians to provide definitive diagnoses in all cases, as the physician’s role is to rule out immediately dangerous conditions rather than provide a definitive diagnosis. Finally, per our research protocol we excluded pregnant and pediatric patients; however, these patients could also benefit from SBD research and future methods should consider including these populations.

## CONCLUSION

This study demonstrates an application of the UMLS to identify symptom-based diagnoses, with the semantic type of “Sign or Symptom” showing high sensitivity and specificity compared to manual review. Automation of this time-intensive process could facilitate large-scale studies on the effects of symptom-based diagnoses or other non-disease-based events associated with an episode of care.

## Supplementary Information



## Figures and Tables

**Table 1:** Frequencies of semantic types among all included emergency department discharges (N = 3642).

Semantic type	n	Percent
Sign or Symptom	1367	37.5%
Disease or Syndrome	916	25%
Injury or Poisoning	643	17.6%
Finding	358	9.8%
Mental or Behavioral Dysfunction	163	4.5%
Pathologic Function	155	4.2%
Acquired Abnormality	20	0.5%
Neoplastic Process	10	0.3%
Anatomical Abnormality	9	0.2%
Body Substance	1	0.03%

**Table 2:** Patient encounter level statistics (N = 3642).

Semantic type	SBD	Not SBD	Total	
Sign or Symptom	TP = 1288	FP = 79	1367	PPV = 0.942
Not Sign or Symptom	FN = 233	TN = 2042	2275	NPV = 0.898
Total	1521	2121	3642	
	Sn = 0.847	Sp = 0.963		

*SBD*, symptom-based diagnosis; *TP*, true positives; *FP, f*alse positives; *TN*, true negatives; *FN*, false negatives; *Sn*, sensitivity; *Sp*, specificity; *PPV*, positive predictive value; *NPV*, negative predictive value.

**Table 3:** Top 10 encounter-level diagnoses with associated ICD-10 codes and ”Concept Unique Identifiers” classified as both “Sign or Symptom” semantic type and symptom-based diagnosis (N=3,642).

ICD-10 code	Discharge diagnosis	CUI code	Semantic type	SBD	n
R07.9	Chest pain, unspecified type	C0008031	Sign or Symptom	Yes	153
R51	Nonintractable headache, unspecified chronicity pattern, unspecified headache type	C0018681	Sign or Symptom	Yes	61
R10.9	Abdominal pain, unspecified abdominal location	C0000737	Sign or Symptom	Yes	43
R10.84	Generalized abdominal pain	C0344304	Sign or Symptom	Yes	38
R51	Acute nonintractable headache, unspecified headache type	C0018681	Sign or Symptom	Yes	37
R06.02	Shortness of breath	C0013404	Sign or Symptom	Yes	35
R07.89	Chest wall pain	C0029537	Sign or Symptom	Yes	28
R42	Dizziness	C0476206	Sign or Symptom	Yes	25
R05	Cough	C0010200	Sign or Symptom	Yes	23
R21	Rash	C0015230	Sign or Symptom	Yes	23

*ICD-10*, International Classification of Diseases, 10th ed; *SBD*, symptom-based diagnosis; *CUI*, concept unique identifier.

**Table 4:** Top 10 encounter-level diagnoses with associated ICD-10 codes and “Concept Unique Identifiers” classified as “Sign or Symptom” semantic type but not as symptom-based diagnosis (N = 3642).

ICD-10 code	Discharge diagnosis	CUI code	Semantic type	SBD	n
R56.9	Seizure (CMS/HCC)	C0036572	Sign or Symptom	No	16
K59.00	Constipation, unspecified constipation type	C0009806	Sign or Symptom	No	8
M79.1	Musculoskeletal pain	C0231528	Sign or Symptom	No	5
R42	Postural dizziness with presyncope	C0476206	Sign or Symptom	No	5
G89.18	Post-op pain	C2875361	Sign or Symptom	No	4
R46.89	Suicidal behavior without attempted self-injury	C0478141	Sign or Symptom	No	4
M62.838	Muscle spasm	C2895804	Sign or Symptom	No	3
R55	Vasovagal syncope	C0039070	Sign or Symptom	No	3
G89.18	Post-operative pain	C2875361	Sign or Symptom	No	2
R56.9	Seizures (CMS/HCC)	C0036572	Sign or Symptom	No	2

*ICD-10*, International Classification of Diseases, 10th ed; *SBD*, symptom-based diagnosis; *CUI*, concept unique identifier; *CMS/HCC*, Centers for Medicare and Medicaid Services/hierarchical condition category.

**Table 5:** Top 10 encounter-level diagnoses with associated ICD-10 codes and “Concept Unique Identifiers” classified as symptom-based diagnosis but not as “Sign or Symptom” semantic type (N = 3,642).

ICD-10 code	Discharge diagnosis	CUI code	Semantic type	SBD	n
M25.571	Acute right ankle pain	C3531698	Finding	Yes	17
K08.89	Pain, dental	C0029790	Disease or Syndrome	Yes	15
R00.2	Palpitations	C0030252	Finding	Yes	15
R33.9	Urinary retention	C0080274	Finding	Yes	14
K62.5	Rectal bleeding	C0019081	Pathologic Function	Yes	9
R31.9	Hematuria, unspecified type	C0018965	Disease or Syndrome	Yes	9
M79.89	Leg swelling	C0477668	Disease or Syndrome	Yes	8
M25.572	Acute left ankle pain	C3531697	Finding	Yes	7
N93.9	Vaginal bleeding	C0495117	Pathologic Function	Yes	7
N89.8	Vaginal discharge	C0029819	Disease or Syndrome	Yes	6

*ICD-10*, International Classification of Diseases, 10th ed*; SBD*, symptom-based diagnosis; *CUI*, concept unique identifier.

**Table 6:** Top 10 encounter-level diagnoses with associated ICD-10 codes and “Concept Unique Identifiers” not classified as either “Sign or Symptom” semantic type or symptom-based diagnosis (N = 3642).

ICD-10 code	Discharge diagnosis	CUI code	Semantic type	SBD	n
W19.XXXA	Fall, initial encounter	C2904005	Injury or Poisoning	No	111
F10.920	Alcoholic intoxication without complication (CMS/HCC)	C2874406	Mental or Behavioral Dysfunction	No	59
R45.851	Suicidal ideation	C0424000	Finding	No	30
L02.91	Abscess	C2888089	Pathologic Function	No	20
N12	Pyelonephritis	C0477743	Disease or Syndrome	No	19
N30.00	Acute cystitis without hematuria	C2902964	Disease or Syndrome	No	19
S09.90XA	Head injury, initial encounter	C2832842	Injury or Poisoning	No	19
S61.219A	Finger laceration, initial encounter	C2849879	Injury or Poisoning	No	18
Y09	Assault	C0004063	Injury or Poisoning	No	17
J06.9	Upper respiratory tract infection, unspecified type	C0264222	Disease or Syndrome	No	16

*ICD-10*, International Classification of Diseases, 10th ed; *SBD*, symptom-based diagnosis; *CUI*, concept unique identifier; *CMS/HCC*, Centers for Medicare and Medicaid Services/hierarchical condition category.

**Table 7:** Discharge diagnosis level statistics (N = 1382).

Semantic type	SBD	Not SBD	Total	
Sign or Symptom	TP = 277	FP = 37	314	PPV = 0.882
Not Sign or Symptom	FN = 92	TN = 976	1068	NPV = 0.914
Total	369	1013	1382	
	Sn = 0.751	Sp = 0.964		

*SBD*, symptom-based diagnosis; *TP*, true positives; *FP, f*alse positives; *TN*, true negatives; *FN*, false negatives; *Sn*, sensitivity; *Sp*, specificity; *PPV*, positive predictive value; *NPV*, negative predictive value.
