# Supplementary material for: Identifying Emergency Department Symptom-Based Diagnoses with the Unified Medical Language System
Source: West J Emerg Med. 2019 Oct 24;20(6):910–7. doi: 10.5811/westjem.2019.8.44230 (PMC6860381; doi:10.5811/westjem.2019.8.44230)
Supplement: Supplementary file 1 [file wjem-20-910-s001.docx]

Appendix: Sample R Code

library(data.table)

library(openxlsx)

### Import file names – RRF files are read directly from UMLS###

MRCONSO_FILE <- c("MRCONSO.RRF")

MRSTY_FILE <- c("MRSTY.RRF")

### ICD10 file should consist of 1 column of codes and 1 column of descriptions ####

ICD10_FILE <- c("###ICD10_FILE###.xlsx")

### Read and map UMLS ICD10 Codes to their Semantic Types ###

MRCONSO<-data.table((read.delim(MRCONSO_FILE,sep="|"))[1:18])

setnames(MRCONSO,c("CUI","LAT","TS","LUI","STT","SUI","ISPREF","AUI","SAUI","SCUI","SDUI","SAB","TTY","CODE","STR","SRL","SUPPRESS","CVF"))

MRSTY <-data.table(read.delim(MRSTY_FILE,sep="|")[1:6])

setnames(MRSTY,c("CUI","TUI","STN","STY","ATUI","CVF"))

ICD10<-data.table(read.xlsx(ICD10_FILE))

setnames(ICD10,c("CODE","Description"))

setkey(ICD10,CODE)

setkey(MRCONSO,CODE)

ICD_MRCONSO<-merge(ICD10,MRCONSO[SUPPRESS=="N"],all.x = T)

setkey(ICD_MRCONSO,CUI)

setkey(MRSTY,CUI)

ICD_MRCONSO_MRSTY <-merge(ICD_MRCONSO,MRSTY,all.x = T)

### Isolates Unique ICD10s, their CUI and Semantic Type ###

CUI_CODES_ST<-unique(ICD_MRCONSO_MRSTY[,c("CUI","CODE","STY")])

setkey(CUI_CODES_ST,CODE)

### Creates final results ###

Results<-merge(ICD10,CUI_CODES_ST,all.x = T)

### Prints Results###

Results
